# Spontaneous Emission Mediated by Moiré Hyperbolic Metasurfaces

**DOI:** 10.3390/nano15030228

**Published:** 2025-01-31

**Authors:** Yuying Liu, Zhanrong Yang, Tongbiao Wang, Jianrong Yang, Tianbao Yu, Qinghua Liao

**Affiliations:** 1 School of Physics and Materials Science, Nanchang University, Nanchang 330031, China; yuying_liu2024@163.com (Y.L.); zhanrongyang@whu.edu.cn (Z.Y.); yutianbao@ncu.edu.cn (T.Y.); lqhua@ncu.edu.cn (Q.L.); 2 School of Physics and Electronic Information, Shangrao Normal University, Shangrao 334001, China; sryangjr@163.com

**Keywords:** spontaneous emission, hyperbolic metasurfaces, twisted photonic structure

## Abstract

We investigate the spontaneous emission of a quantum emitter (QE) placed near the twisted hyperbolic metasurfaces (HMTSs) made of graphene strips. We demonstrate that the spontaneous emission can be enhanced distinctly due to the existence of moiré hyperbolic plasmon polaritons (HPPs) supported by the twisted HMTSs. Moreover, the spontaneous emission decay rate can be efficiently modulated by the chemical potential of graphene, the thickness of the dielectric spacer, and the twist angle between two HMTSs. The maximum spontaneous emission is achieved when topological transition occurs. The spontaneous emission will be enhanced as the thickness of the dielectric spacer increases for most cases. In particular, the twisted HMTSs make it possible to flexibly modify the spontaneous emission through the external field. The findings in this work not only extend past studies of twisted photonic structures but also have important applications in optical sensing and integrated photonics.

## 1. Introduction

Spontaneous emission is a common phenomenon occurring in excited quantum systems that is coupled to the surrounding environment. It was once considered to be an inherent property of atoms until the groundbreaking work of Purcell in 1946 [1]. It is shown that spontaneous emission can be drastically increased when the atom is in close proximity to an objective [1]. The spontaneous emission rate is proportional to the electromagnetic local density of states (LDOS) surrounding the atoms. Therefore, it is able to enhance or inhibit the spontaneous emission by modifying the environment near the atom [2]. Furthermore, photonic crystals [3,4,5] and plasmonic nanostructures [6,7] have been widely used to modify spontaneous emission. Moreover, plenty of artificial new-emerging materials [8,9,10,11,12] and hyperbolic metamaterials [12,13,14,15,16,17,18] and metasurfaces [19,20,21,22,23] have been proposed to modulate spontaneous emission in the past decade.

In recent years, there has been a surge of interest in studying moiré superlattices, inspired by the discovery of correlated insulator behavior [24] and unconventional superconductivity [25] at a magic rotation angle. The strong dependence on the rotation angle results in the birth of “twistronics”. Similarly, moiré electronics inspires the study of their photonic counterparts, namely the twisted photonic structures [26,27,28]. Based on the weak van der Waals force between the layers of two-dimensional materials, it is possible to stack multiple layers together, forming a van der Waals (vdW) heterostructure without the constraint to lattice mismatch [29]. The supported plasmon polaritons can propagate in both layers and strongly couple with each other, resulting in the formation of hybridized polaritons [29]. These hybridized polaritons have been utilized to enhance near-field thermal radiation [30,31]. In addition, the presence of appropriate spacing between the two graphene sheets allows for the formation of interlayer excitons [32,33], namely the electron-hole pairs that occur between different layers. These interlayer excitons significantly affect the spectral characteristics, thereby exerting an influence on spontaneous emission [32,33]. Because of their applications in optical sensing, chiral optics, and new on-chip light sources, twisted photonic structures have become a promising platform to modulate novel light–matter interactions. Recently, the photonic LDOS in twisted graphene moiré superlattices was studied in Ref. [34]. The authors demonstrated that the photonic LDOS can be either enhanced or decreased by an order of magnitude for some special twist angels compared to the monolayer graphene. A hyperbolic metasurface (HMTS) can be constructed by tailoring the monolayer graphene sheet to an array of densely packed graphene strips (GSs) [35]. Such an HMTS can support the extreme topological transitions between elliptical and hyperbolic for surface plasmon propagation [35]. Moreover, when an HMTS is laid upon another one with a small twist angle, the moiré HMTSs with exotic optical properties can be obtained [36]. Moiré hyperbolic plasmons can be excited in such a twisted photonic structure. The variation in twist angle can lead to the hyperbolic-to-elliptical topological transition and flat band features with low-loss field canalization [36]. Therefore, the twisted HMTSs have a great advantage for tailoring the photonic LDOS and then for spontaneous emission.

In this work, we investigate the spontaneous emission of a quantum emitter (QE) in the proximity of a twisted HMTS made of graphene strips. The spontaneous emission rate can be enhanced evidently due to the coupling between the radiation of QE and moiré hyperbolic plasmons. We find that the Purcell factor varies with the twist angle and the maximum Purcell factor can be achieved when the topological transition occurs. The spontaneous emission almost increases with the increase in the thickness of the dielectric spacer between two HMTS. In addition, the effect of chemical potential of graphene on the spontaneous emission provides an efficient method to dynamically control the spontaneous emission.

## 2. Theoretical Model

**Figure 1:**
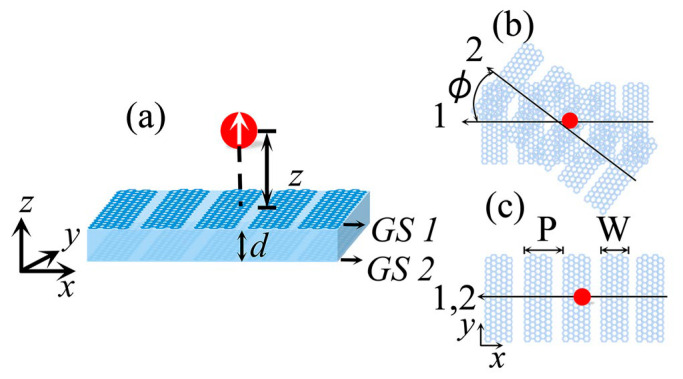
(**a**) The schematic of a QE placed near the surface of the twisted HMTSs with distance z. The thickness of spacer between two HMTSs is d. (**b**) The top view of the system with a twist angle ϕ with respect to the *x*-axis. (**c**) The top view of the system without twist between HMTSs. W and P are the strip width and period, respectively.

A QE placed on the top of the twisted HMTSs with a distance z is illustrated in Figure 1a. Two HMTSs made of GSs are denoted as GS1 and GS2, respectively. It has been demonstrated that the array of densely packed GSs behave as HMTSs [35]. The extreme topological transitions—from closed elliptical to open hyperbolic—for surface plasmon propagation have been explored [35]. The strip width and period of the HMTS are denoted as W and P, respectively. We define f=W/P as the filling fraction. The optical conductivity of the HMT can be analytically obtained by the effective medium theory (EMT), which is given as [35]

(1) $$\sigma_{eff}=[\begin{array}{cc} \sigma_{xx}^{eff} & 0\\ 0 & \sigma_{yy}^{eff} \end{array}]=\left[\begin{array}{cc} \frac{P\sigma \sigma_{C}}{W\sigma_{C}+G\sigma } & 0\\ 0 & \frac{W}{P}\sigma \end{array}\right],$$

where $\sigma_{C}=-i\omega \varepsilon_{0}P/\left(\pi \mathrm{lnn}\{\csc[0.5\pi (1-f)]\}\right)$ is an equivalent conductivity associated with the near-field coupling between adjacent strips obtained using an electrostatic approach [35]. $\sigma =\sigma_{D}+\sigma_{I}$ is the optical conductivity of graphene, where $\sigma_{D}$ and $\sigma_{I}$ denote the intraband and interband conductivity, expressed, respectively, as [37]

(2) $$\begin{array}{c} \sigma_{D}=\frac{i}{\omega +i/\tau }\frac{2e^{2}k_{B}T}{\pi \hbar^{2}}ln\left[2cosh\frac{\mu }{2k_{B}T}\right],\\ \sigma_{I}=\frac{e^{2}}{4\hbar }\left[G\left(\frac{\hbar \omega }{2}\right)+i\frac{4\hbar \omega }{\pi }\int_{0}^{\infty }\frac{G\left(\xi \right)-G(\hbar \omega /2)}{{\left(\hbar \omega \right)}^{2}-4\xi^{2}}d\xi \right], \end{array}$$

where $G\left(\xi \right)=\sinh(\xi /k_{B}T)/\left[\cosh\left(\mu /k_{B}T\right)+cosh(\xi /k_{B}T)\right]$ and μ is the chemical potential that can be altered by the gate voltage or external field. e is the electron charge, ℏ is the Planck constant, $k_{B}$ is the Boltzmann constant, and T is the temperature that is set to 300 K in this work. $\tau =10^{-13}\;s$ is the electron scattering lifetime. The thickness of the dielectric spacer between GS1 and GS2 is d. Although two HMTSs should be separated by a thin dielectric film, such as SiO_2_ or PMMA, we assume that the dielectric constant of the spacer layer is 1.0 without having an important effect on the final results.

**Figure 2:**
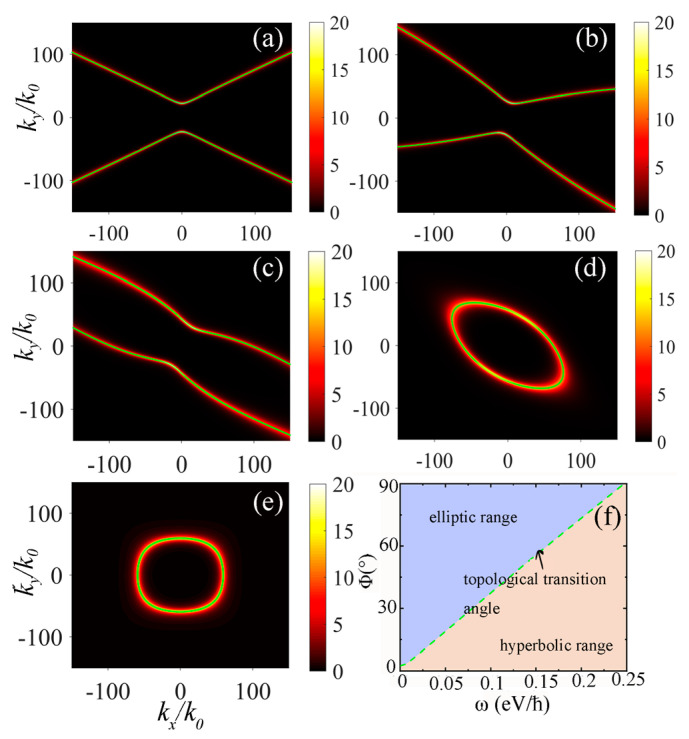
The imaginary parts of reflection coefficients about moiré HMTSs with twist angles of (**a**) $\phi =0^{{}^{\circ}}$, (**b**) $\phi =30^{{}^{\circ}}$, (**c**) $\phi =60^{{}^{\circ}}$, (**d**) $\phi =80^{{}^{\circ}}$, and (**e**) $\phi =90^{{}^{\circ}}$. The parameters are set to d=1.0 nm, μ=0.4 eV, W=6 nm, P=10 nm, and $\varepsilon_{2}=1$. The angular frequency is 0.15 eV/ℏ. The green lines denote the dispersion relation. (**f**) Topological transition regions as a function of frequency and twist angles. The green dashed line represents the topological transition angle.

Axes 1 and 2 are, respectively, perpendicular to the strips of GS1 and GS2, as shown in Figure 1b. In this work, the GS1 is fixed in xy plane, while GS2 can be twisted with an angle ϕ with respect to the *x*-axis, as shown in Figure 1b. In this scenario, the normalized spontaneous emission (i.e., the Purcell factor) for the perpendicularly polarized electric dipole can be expressed as [38]

(3) $$\frac{\Gamma }{\Gamma_{0}}=1+\frac{6\pi }{\left|{\vec{\mu }}_{p}\right|k_{0}}Im\left\{{\vec{\mu }}_{p}\cdot \left[\hat{G}\left({\vec{r}}_{0},{\vec{r}}_{0},\omega \right)\right]\cdot {\vec{\mu }}_{p}\right\},$$

where ${\vec{\mu }}_{p}$ is the unit vector aligned with the orientation of the dipole moment, ${\vec{r}}_{0}$ is the source position, and $k_{0}=\omega /c$ is the wavenumber in vacuum. Green’s tensor $\hat{G}({\vec{r}}_{0},{\vec{r}}_{0},\omega )$ of the system at the source positon can be expressed using the following formula [38]:

(4) $$\hat{G}\left({\vec{r}}_{0},{\vec{r}}_{0},\omega \right)=\frac{i}{8\pi^{2}}\int_{-\infty }^{\infty }\int_{-\infty }^{\infty }\left(r_{ss}{\vec{M}}_{ss}+r_{sp}{\vec{M}}_{sp}+r_{ps}{\vec{M}}_{ps}+r_{pp}{\vec{M}}_{pp}\right)e^{2ik_{z}z}dk_{x}dk_{y},$$

where ${\vec{M}}_{mn}(m,n=s,p)$ is the matrix element that can be obtained from Refs. [24,25]. $r_{ss}$ and $r_{pp}$ correspond to the reflection coefficients for the s- and p-polarized electromagnetic waves, respectively. $r_{sp}$ and $r_{ps}$ are the cross-reflection coefficients. These reflection coefficients are obtained with the help of a 4 × 4 T-matrix formalism, and a detailed derivation process can be found in Refs. [25,39,40,41]. In our calculations, we assume that the refractive index of the dielectric spacer is 1.0. For a multilayer GS system, the T-matrix can be extended to encompass any number of layers by multiplying the T-matrix corresponding to each GS layer, so we can obtain the reflection matrix for the multilayer GS system. By employing the method provided in the previous publications, the reflection coefficients can be expressed as [42]

(5) $$r_{pp}=\frac{T_{\left(2,1\right)}T_{\left(3,3\right)}-T_{\left(2,3\right)}T_{\left(3,1\right)}}{T_{\left(1,1\right)}T_{\left(3,3\right)}-T_{\left(1,3\right)}T_{\left(3,1\right)}},\;r_{ps}=\frac{T_{\left(4,1\right)}T_{\left(3,3\right)}-T_{\left(4,3\right)}T_{\left(3,1\right)}}{T_{\left(1,1\right)}T_{\left(3,3\right)}-T_{\left(1,3\right)}T_{\left(3,1\right)}},\;r_{sp}=\frac{T_{\left(1,1\right)}T_{\left(2,3\right)}-T_{\left(1,3\right)}T_{\left(2,1\right)}}{T_{\left(1,1\right)}T_{\left(3,3\right)}-T_{\left(1,3\right)}T_{\left(3,1\right)}},\;r_{ss}=\frac{T_{\left(1,1\right)}T_{\left(4,3\right)}-T_{\left(1,3\right)}T_{\left(4,1\right)}}{T_{\left(1,1\right)}T_{\left(3,3\right)}-T_{\left(1,3\right)}T_{\left(3,1\right)}},$$

where $T_{\left(i,j\right)}(i,j=1,2,3,4)$ represents the elements of the T-matrix components. Then, we can obtain the reflection coefficients of the p-polarized evanescent waves in the twisted HMTS system. The parameters of the system are set to d=1.0 nm, $\varepsilon_{2}=1$, W=6 nm, and P=10 nm. The chemical potential of graphene for two HMTSs are set to μ=0.4 eV. In Figure 2, we show the imaginary parts of the reflection coefficient $r_{pp}$ in phase space for different twist angles, where the green lines represent the dispersion relation. The twist angles in Figure 2a–e are set to 0°, 30°, 60°, 80°, and 90°, respectively. The angular frequency of the incident electromagnetic wave is set to ω=0.15 eV/ℏ. In Figure 2a–e, we observe that as the twist angle increases, the dispersion relation curve rotates around the origin. Moreover, it is noteworthy that the dispersion curve exhibits a hyperbolic shape for the twist angle ranging from 0° to 30°. Then, the dispersion curve gradually translates into a closed ellipse as the twist angle continues to increase. In other words, the dispersion curve undergoes a topological transition from a hyperbolic to an elliptical shape when the twist angle reaches a certain value. In order to obtain the transition angle when the topological transition occurs, we show the dependence of the topological transition angle on frequency by using the method proposed in Ref. [36] in Figure 2f. We can see that the corresponding topological transition angle is 55.6° for the angular frequency 0.15 eV/ℏ. The topological transition of the dispersion curve with the twist angle lies in the coupling effect caused by weak van der Waals forces between two HMTSs. This coupling allows the twisted HMTSs to form an effective heterostructure, enabling two anisotropic HMTSs to couple with each other to form a moiré hyperbolic metasurface [25,36]. Moreover, the coupling between the hyperbolic plasmons existing in each HMTS forms the hybrid topological polaritons.

## 3. Results and Discussions

**Figure 3:**
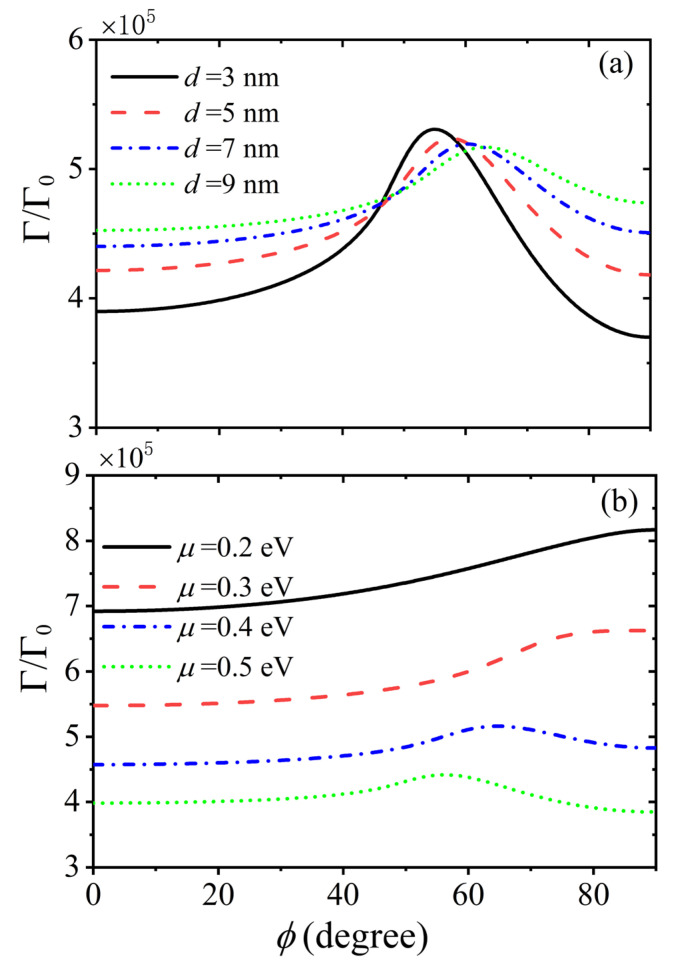
The Purcell factor of the QE as a function of twist angle for different (**a**) thicknesses of dielectric spacer with a fixed chemical potential of 0.4 eV and (**b**) the chemical potential of graphene with a fixed thickness of a dielectric spacer of 10 nm. The distance z is fixed at 10 nm. The period P is 10 nm and the filling factor is f=0.6 for the moiré HMTSs. The transition frequencies are set to 0.15 eV/ℏ.

First, we show the dependence of the Purcell factor of a QE on the twist angle for different thicknesses of the dielectric spacer in Figure 3a. The distance between the QE and the moiré HMTSs is fixed at z=10 nm. The width of GS is W=6 nm and period is P=10 nm. The chemical potential of graphene is μ=0.4 eV, and the dielectric constant is set to $\varepsilon_{2}=1$ in the calculations. In Figure 3a, we can see that the Purcell factor increases with the twist angle, and they will decrease as the twist angle continues to increase after reaching the maximum values for all considered thicknesses of dielectric spacer. With the help of Figure 2f, we find that the occurrence of the maximum Purcell factor corresponds to the twist angle where the topological transition takes place. The directional canalized plasmon polariton can be supported in the twisted HMTSs, which result in the enhancement of the spontaneous emission. Additionally, according to Ref. [36], as the distance d between the two HMTSs increases, the magic angle of topological transition increases slightly. Therefore, we can see that the maximum Purcell factor moves toward a larger twist angle as the thickness of the dielectric spacer increases. Furthermore, when the twist angle is far from the magic angle of topological transition (less than $40^{{}^{\circ}}$ or larger than $60^{{}^{\circ}}$), the Purcell factor increases with the thickness of dielectric spacer for the given twist angle. However, in the topological transition range, a larger Purcell factor is obtained for a thinner dielectric spacer. When the twist angle is much less than the topological transition angle, the hybrid HPPs from each HMTS dominate the spontaneous emission.

The thinner dielectric spacer results in a weaker hybridization; then, a smaller Purcell factor is obtained. The similar phenomenon can be analyzed when the twist angle is much larger than the magic angle, where the hybrid surface plasmons are excited. Near the region of topological transition, the surface plasmons are canalized and propagate in a fixed direction, which will lead to a large Purcell factor. The dependence of the Purcell factor on the twist angle for different chemical potentials is shown in Figure 3b. For the chemical potential of 0.2 eV, the Purcell factor increases as the twist angle increases. Compared with that of the system without twist, the spontaneous emission enhances remarkably when the twist angle reaches $90^{{}^{\circ}}$. However, for the other considered chemical potentials, the variation in spontaneous emission with the twist angle is not monotonous. The maximum Purcell factors can be obtained at special twist angles for these chemical potentials. This means that the magic angle for the occurrence of the topological transition is also dependent on the chemical potential of graphene.

**Figure 4:**
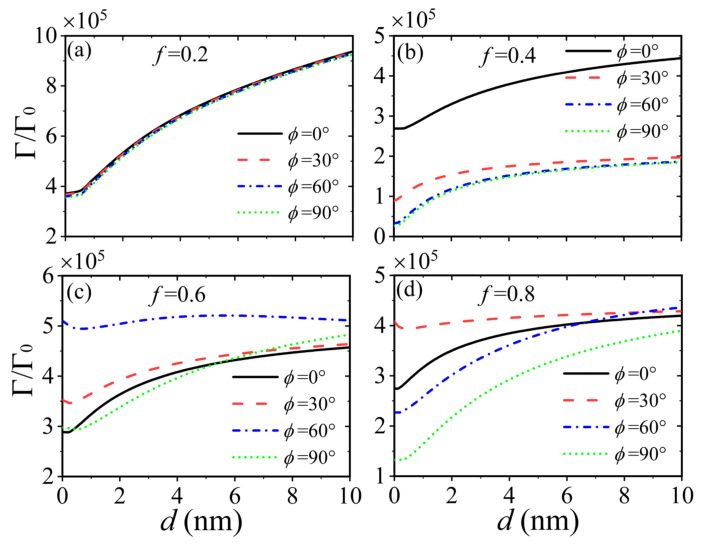
The Purcell factor of the QE as a function of the thickness of the dielectric spacer for different twist angles. The filling fractions are 0.2, 0.4, 0.6, and 0.8 from (**a**–**d**), respectively. The twist angles in (**b**–**d**) are the same as those in (**a**). The distance z is set to 10 nm, the period P is 10 nm, the chemical potential is 0.4 eV, and the transition frequency is 0.15 eV/ℏ.

In Figure 4, we display the dependence of the Purcell factor as a function of the thickness of the dielectric spacer for different twist angles. The filling fractions of the HMTSs are set to 0.2, 0.4, 0.6, and 0.8 in Figure 4a–d, respectively. It is seen that the Purcell factor increases evidently with the thickness of dielectric spacer for any given filling fraction except the case with filling fraction of 0.4 and twist angel of $60^{{}^{\circ}}$, as shown in Figure 4c. For the filling fraction of 0.2, the Purcell factor near the twisted HMTSs with d=10 nm is twice the value of that near the twisted HMTSs with d=1 nm, as shown in Figure 4a. However, the spontaneous emission is not sensitive to the twist angle for the smaller filling fraction (f=0.2). For a filling fraction of f=0.4, the spontaneous emission decreases obviously as the twist angle increases at a given dielectric spacer, as shown in Figure 4b. In addition, the Purcell factor is much larger for the system without twisting than those with twisting. The spontaneous emission of a QE near the twisted HMTSs with a filling fraction of f=0.6 is displayed in Figure 4c. We can see that the Purcell factor does not keep increasing as the thickness of dielectric spacer increases for the twist angle of $60^{{}^{\circ}}$. Moreover, the Purcell factor for the twist angle of $60^{{}^{\circ}}$ is always larger than those for the other three twist angles. For the filling fraction of f=0.8, it is seen that the thickness of the dielectric spacer has more effect on spontaneous emission for the twist angle of $90^{{}^{\circ}}$ than on that for the twist angle of $0^{{}^{\circ}}$.

**Figure 5:**
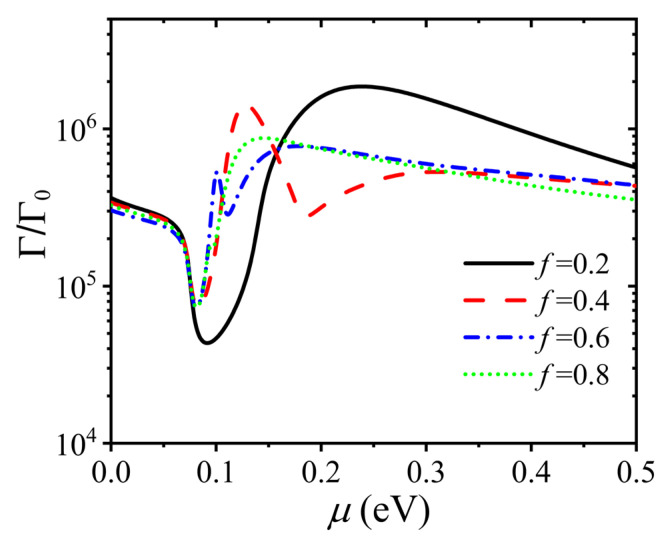
The Purcell factor of the QE as a function of chemical potential for different filling factors. The distance z is set to 10 nm, the period P is 10 nm, the thickness of the spacer is 10 nm, and the transition frequency is 0.15 eV/ℏ.

**Figure 6:**
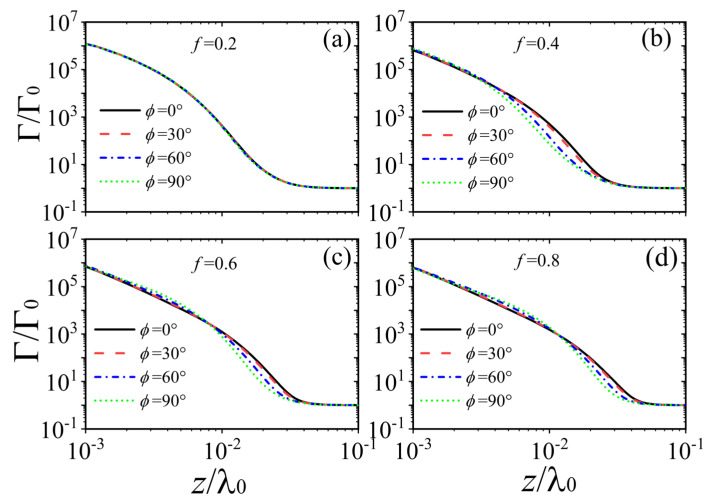
The Purcell factor as a function of distance z for different twist angles. The filling fractions in (**a**–**d**) are f=0.2, f=0.4, f=0.6, and f=0.8, respectively. The twist angles in (**b**–**d**) are the same as those used in (**a**). The period is fixed at P=10 nm, and the other parameters are the same with those used in Figure 2.

In Figure 5, we investigate the effect of the chemical potential of graphene on the spontaneous emission since it plays an important role in the optical characteristic of the moiré HMTSs. We display the dependence of the Purcell factor on the chemical potential for different filling fractions. The transition frequency of QE is still set to 0.15 eV/ℏ. The distance between the QE and the twisted HMTSs is set to d=10 nm. An intriguing phenomenon can be observed in our considered range of chemical potential. For the cases of f=0.2 and f=0.8, there are one peak and one dip corresponding to the largest and the smallest spontaneous emission rate in the Purcell factor curves, as shown in Figure 6. However, for the case of f=0.4, there are one peak and two dips in the Purcell factor curve. Furthermore, there are two peaks and two dips in the Purcell factor curve for the case of f=0.6. In addition, the spontaneous emission is always larger than those of the other cases when the chemical potentials are from 0 to 0.05 eV and from 0.15 to 0.5 eV. The maximum Purcell factor is nearly one hundred times larger than the minimum one for the case of f=0.2, which can be seen from the black solid line in Figure 5. Therefore, the spontaneous emission of a QE near the twisted HMTSs can be actively modified by the chemical potential controlled by the external field.

Finally, we investigate the effect of the filling fraction on the spontaneous emission. Figure 6 depicts the relationship between the Purcell factor of a single QE and the distance z for different twist angles. The filling fractions of graphene strip are set to 0.2, 0.4, 0.6, and 0.8 in Figure 6a–d, respectively. Four special twist angles with values of 0°, 30°, 60°, and 90° are all considered in each subfigure. The results demonstrate that the spontaneous emission of the QE in proximity to the moiré HMTSs experiences an obvious decrease as the distance increases. Remarkably, the maximum values of the Purcell factor (approximately $10^{6}$) are achieved at the closest distance $z=10^{-3}\lambda_{0}$. However, as the distance increases to $z=10^{-1}\lambda_{0}$, the Purcell factor decreases to about 1.0, indicating that the effect of moiré HMTSs on the spontaneous emission can be neglected when the QE is far from the sample. Therefore, the radiation emitted from the QE will dissipate into free vacuum, so the topological polaritons cannot be excited efficiently. In addition, we can see that in Figure 6a, the spontaneous emission is not sensitive to the twist angle for the filling fraction f=0.2, which is similar to that in Figure 4a. This is due to the fact that the coupling between two neighboring HMTSs is weak for the smaller filling fraction. However, when the filling fraction increases, the coupling between neighboring strips increases because of the decrease in the separation between adjacent strips. Therefore, the effect of twist angle on the spontaneous emission becomes stronger and stronger, as shown in Figure 6b. When the distance is near $z=10^{-2}\lambda_{0}$, the dependence of the spontaneous emission with respect to distance declines faster for the larger twist angle than for the smaller twist angle. A similar phenomenon can also be observed in Figure 6c,d.

## 4. Conclusions

Because twisted photonic structures have become a promising platform to modulate light–matter interactions, they may lead to many new phenomena in future studies of spontaneous emission. We have studied the spontaneous emission of a QE near the twisted HMTSs. We found that twisted HMTSs can help to enhance the spontaneous emission of the QE significantly. The spontaneous emission rate can be modulated flexibly by the twist angle, the thickness of the dielectric spacer, and the chemical potential of graphene. As the twist angle increases, the spontaneous emission rate decreases more rapidly with the distance for the filling fraction of 0.4. The twist angle has an important effect on spontaneous emission, especially for the twisted HMTSs with a thinner dielectric spacer and smaller chemical potential. The maximum spontaneous emission can be obtained when the topological transition occurs, which results in the canalized plasmon polaritons. However, reducing the chemical potential will shift the maximum Purcell factor toward a larger twist angle. For the smaller filling fraction, changing the twist angle has little effect on spontaneous emission. We believe the results obtained in this work will extend the application of twisted photonic structures and provide a new avenue to control spontaneous emission.

## Data Availability

The data that support the findings of this study are available upon reasonable request from the corresponding author.
